# Management of sudden sensorineural hearing loss among primary care physicians in Canada: a survey study

**DOI:** 10.1186/s40463-021-00498-x

**Published:** 2021-04-01

**Authors:** Benjamin Ng, Matthew G. Crowson, Vincent Lin

**Affiliations:** 1 Department of Otolaryngology-Head & Neck Surgery, Sunnybrook Health Sciences Centre, 2075 Bayview Avenue, Toronto, Ontario M4N 3M5 Canada; 2 Department of Otolaryngology-Head & Neck Surgery, Massachusetts Eye & Ear, Boston, MA USA; 3 Department of Otolaryngology-Head & Neck Surgery, Harvard Medical School, Boston, MA USA

**Keywords:** sudden hearing loss, sudden sensorineural hearing loss, Tuning fork, Weber test, Rinne test

## Abstract

**Background:**

Sudden Sensorineural Hearing Loss (SSNHL) is a medical emergency requiring immediate attention as delayed treatment can lead to permanent and devastating consequences. Primary care physicians are likely the first to be presented with SSNHL and therefore have the crucial role of recognizing it and initiating timely and appropriate management. The aim of this study was to gain insight into the current knowledge and practice trends pertaining to the diagnosis and management of SSNHL among family physicians in Canada.

**Methods:**

An 18-question survey targeting Canadian family physicians was marketed through two, physician-only discussion groups on the social media platform Facebook. Responses were collected between August 1st and December 22nd 2019 then aggregated and quantified.

**Results:**

52 family physicians submitted responses. 94.2% (*n* = 49) reported that in their practice, unilateral SSNHL warrants urgent referral to otolaryngology and 84.6% (*n* = 44) reported that unilateral sudden-onset hearing loss warrants urgent referral for audiological testing. 73.1% of participants (*n* = 38) reported that they would attempt to differentiate between conductive and sensorineural hearing loss if presented with unilateral, acute or sudden-onset hearing loss. 61.5% (*n* = 32) would rely on tuning fork tests to inform management decisions, as compared to 94.2% (*n* = 49) relying on case history and 88.5% (*n* = 46) on otoscopy. 76.9% (*n* = 40) would prescribe corticosteroids if presented with confirmed, unilateral SSNHL.

**Conclusion:**

The majority of family physicians in the study would make appropriate referral and treatment decisions in the management of SSNHL, understanding it is a medical emergency. Tuning fork tests are under-utilized for informing management decisions compared to other means of differentiating conductive and sensorineural hearing loss. Further research is needed to understand why some family physicians do not prescribe corticosteroids for treatment of SSNHL, which may then identify any gaps in knowledge or inform improvements in clinical protocol.

**Graphical abstract:**

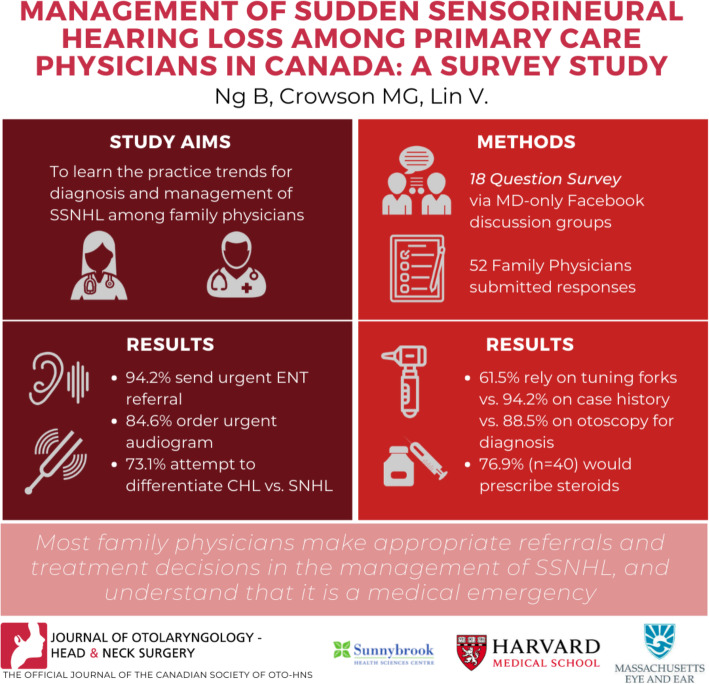

**Supplementary Information:**

The online version contains supplementary material available at 10.1186/s40463-021-00498-x.

## Background

Sudden Sensorineural Hearing Loss (SSNHL) is a medical emergency that can lead to permanent and devastating consequences if left untreated. In the literature, SSNHL is often defined as the rapid onset of hearing loss ≥30 dB HL across three contiguous frequencies, in 72 h or less [1]. The hearing loss can develop either instantaneously or over the course of several hours within this time period [2]. Patients almost always report aural fullness and tinnitus as accompanying symptoms, with some experiencing vertigo as well [3–8]. SSNHL is typically unilateral, with bilateral involvement occurring less than 2% of the time [9]. In the United States, the incidence of SSNHL ranges from 5 to 27 per 100,000 people, with 60,000 new cases every year [2, 10].

SSNHL is often idiopathic in nature but several etiologies have been proposed, including viral infection, vascular impairment, autoimmune disorder, otologic disease and retrocochlear pathology. The viral theory suggests SSNHL occurs when the cochlea or cochlear nerve is compromised by either an invading virus or reactivation of a latent neurotropic virus in the inner ear. Alternatively, a systemic viral infection can trigger an immune-mediated response or activation of the cellular stress pathway in the cochlea [11]. SSNHL can also be a consequence of impaired blood flow to the cochlea resulting from acute vascular hemorrhage [12, 13], embolic occlusion [14], vascular disease [15], vasospasm [16] or hyperviscosity [17, 18]. Several autoimmune disorders, including Cogan’s Syndrome [19, 20], multiple sclerosis [21, 22], rheumatoid arthritis [23], systemic lupus erythematosus [24, 25] and Behçet Syndrome [26, 27] can give rise to SSNHL. Sudden hearing loss is also characteristic of Meniere’s disease [2, 28–30], and vestibular schwannoma [31–35].

A number of factors that correlate with hearing recovery outcomes in SSNHL have been identified. Degree and configuration of the hearing loss at time of onset are commonly reported to have prognostic value. Those initially presenting with a greater degree of hearing loss are likely to have poorer recovery [2, 36–38]. Flat or down-sloping audiogram shape are negative prognostic indicators while low-frequency or mid-frequency hearing loss configurations are associated with higher rates of recovery [8, 36–40]. Presence of vertigo at the time of presentation is also associated with a less favourable prognosis [2, 37, 38, 41]. In terms of demographic factors, older age has consistently been documented to be correlated with lower rates of hearing recovery and poorer threshold gains [2, 8, 36–38, 42].

The current standard for initial treatment of SSNHL is a tapered course of oral corticosteroids [43, 44]. Previous studies have found that the sooner treatment is initiated, the greater likelihood of recovery [3, 8, 29, 45, 46]. Some have even demonstrated that patients who begin treatment within seven days of onset, have significantly better hearing outcomes than those who start later [2, 47]. The importance of treating SSNHL in a timely manner can be appreciated when considering the challenges associated with unilateral hearing loss, such as poor speech understanding in background noise and compromised sound localization, as well as the psychosocial impact on affected individuals [7, 43, 48]. In addition to hearing recovery, successful treatment of SSNHL usually results in an improvement of the concomitant tinnitus [5, 43]. This is significant as persistent tinnitus following SSNHL is associated with higher levels of emotional distress and poorer quality of life, and can pose a significant psychological and economic burden [4, 43, 49, 50].

Primary care physicians (PCPs) are likely to be the first point of contact for patients seeking medical attention for SSNHL and therefore have the crucial role of recognizing potential cases and initiating appropriate management quickly. At present, there is a lack of research that explores the management practices of PCPs in Canada when presented with sudden hearing loss. The specific aim of this study was to gain insight into the current knowledge and practice trends pertaining to the diagnosis and management of SSNHL among Canadian family physicians.

## Methods

### Survey design

An 18-question forced answer choice survey targeting family physicians in Canada was marketed through two private, physicians-only discussion groups on the social media platform, Facebook. The two groups were, “First 5 Years in Family Practice – Canada” and “The Canadian Doctors Lounge,” which contained 4765 and 2217 members, respectively, at the study’s commencement. A link to the electronic survey and a brief description of the study was posted in each of the group discussion forums to solicit participation. To ensure responses were only obtained from the target population, the Study Information Letter presented at the beginning of the survey explicitly stated that only participation from family physicians in Canada was being sought. The electronic survey link was available from August 1st through December 22nd 2019. The survey was hosted using Google Forms, the free online secure-link survey platform. Approval for this study was granted by the Sunnybrook Health Sciences Centre Research Ethics Board.

The first twelve survey questions were designed to assess knowledge and practice trends in the diagnosis and management of SSNHL [**see Survey Copy**]. The last six questions were used to obtain demographic information about the participants. No information that could be used to identify participants was collected.

### Statistical analysis

Responses were automatically aggregated and quantified by Google Forms. Additionally, all data was tabulated in Microsoft Excel so that responses from each individual participant could be reviewed for any inconsistencies.

## Results

### Participant demographics

In total, 52 family physicians completed the survey. The majority had practiced fewer than five years (67.3%; *n* = 35) with only two participants having practiced more than 20 years (3.8%). Most participants reported practicing primarily in Ontario (69.2%; *n* = 36) with the remaining responses representing family physicians in Manitoba, Alberta, British Columbia, Newfoundland & Labrador, Nova Scotia and Quebec. Half the participants primarily provided care in urban areas (50%), 12 in suburban (23.1%) and 14 in rural (26.9%). Half the participants reported primarily practicing in Non-Academic Group or Team settings (50%) but nine in Academic Group or Team settings (17.3%) and nine in Urgent Care or Emergency Departments (17.3%) (Table 1).

**Table 1:** Study participant demographics

	n	%
Years in practice		
Less than 5 years	35	67.3
5–10 years	12	23.1
11–15 years	3	5.8
16–20 years	0	0
More than 20 years	2	3.8
The geographical region in which you primarily serve patients would be considered:		
Urban	26	50
Suburban	12	23.1
Rural	14	26.9
The setting in which you primarily practice would be considered:		
Solo practice	4	7.7
Walk-in clinic	4	7.7
Urgent Care or Emergency Department	9	17.3
Non-Academic Group or Team	26	50
Academic Group or Team	9	17.3
In the past 6 months, number of patients presenting to you with complaints of unilateral, acute or sudden-onset hearing loss, not as a result of cerumen impaction:		
Fewer than 5	46	88.5
5–10	6	11.5
11–15	0	0
16–20	0	0
More than 20	0	0
In the past 12 months, typical wait-time for your patients to be seen by an otolaryngologist, referred for unilateral, sudden-onset hearing loss:		
1 week or less	16	30.8
1–4 weeks	28	53.8
1–3 months	4	7.7
3–6 months	3	5.8
Greater than 6 months	1	1.9
Greater than 12 months	0	0
The province/territory in which you primarily practice:		
British Columbia	2	3.8
Alberta	5	9.6
Saskatchewan	0	0
Manitoba	3	5.8
Ontario	36	69.2
Quebec	2	3.8
New Brunswick	0	0
Nova Scotia	2	3.8
Prince Edward Island	0	0
Newfoundland and Labrador	2	3.8
Yukon	0	0
Northwest Territories	0	0
Nunavut	0	0

Of the 52 participants, the vast majority reported fewer than five patients presenting with complaints of unilateral, acute or sudden-onset hearing loss, not as a result of cerumen impaction, in the past six months (88.5%; *n* = 46). The remaining six participants reported encountering only 5–10 such patients in the past six months (11.5%). More than half of the family physicians who participated in the survey reported that 1–4 weeks was the typical wait-time for a patient, referred for unilateral, sudden-onset hearing loss, to be seen by an otolaryngologist (53.8%; *n* = 28). 16 reported the typical wait-time to be one week or less (30.8%) (Table 1).

### Knowledge and practice trends

Only 15 of the 52 participants correctly defined SSNHL as hearing loss that can develop within a period of 72 h (28.8%). Most understood it as developing in less than 24 h (40.4%; *n* = 21). Eight defined SSNHL as hearing loss that develops within 48 h (15.4%) and eight defined it as developing over a period of seven days (15.4%) (Table 2).

**Table 2:** Aggregated responses to survey questions regarding diagnosis and management of SSNHL

	n	%
According to your definition, SSNHL is defined as hearing loss that can develop over a period of:		
Less than 24 h	21	40.4
48 h	8	15.4
72 h	15	28.8
7 days	8	15.4
Which of the following referrals do you make upon presentation of suspected unilateral SSNHL? Check all that apply.		
Labwork	9	17.3
CT	8	15.4
MRI	9	17.3
Audiologic Evaluation	46	88.5
Otolaryngology consultation	42	80.8
Neurology consultation	2	3.8
Emergency Department	10	19.2
When presented with unilateral, acute or sudden-onset hearing loss, do you attempt to differentiate between conductive and sensorineural hearing loss?		
Yes	38	73.1
No	14	26.9
Do you use tuning fork tests to differentiate between conductive and sensorineural hearing loss?		
Yes	33	63.5
No	19	36.5
Do you feel comfortable interpreting a formal audiogram to differentiate between a conductive hearing loss and sensorineural hearing loss?		
Yes	16	30.8
No	36	69.2
As a family physician, which of the following pharmacologic agents do you prescribe as treatment when presented with suspected unilateral SSNHL, prior to confirmation with audiological testing? Check all that apply.		
Corticosteroids	39	75
Antivirals	6	11.5
Thrombolytics	0	0
Vasodilators	0	0
Other (e.g. antibiotics)	0	0
None of the above	1	1.9
I do not prescribe any pharmacologic agents when presented with suspected SSNHL	12	23.1
Which of the following topics do you include as part of counselling to patients presenting with unilateral SSNHL? Check all that apply.		
Possible causes	28	53.8
Available treatment options and associated risks/benefits	26	50
Impact on Quality of Life	13	25
Rehabilitation options (e.g. hearing aids)	6	11.5
None of the above	1	1.9
I do not counsel patients presenting with SSNHL as I am rarely certain of the diagnosis upon initial presentation	22	42.3

SSNHL: sudden sensorineural hearing loss

**Fig. 1:**
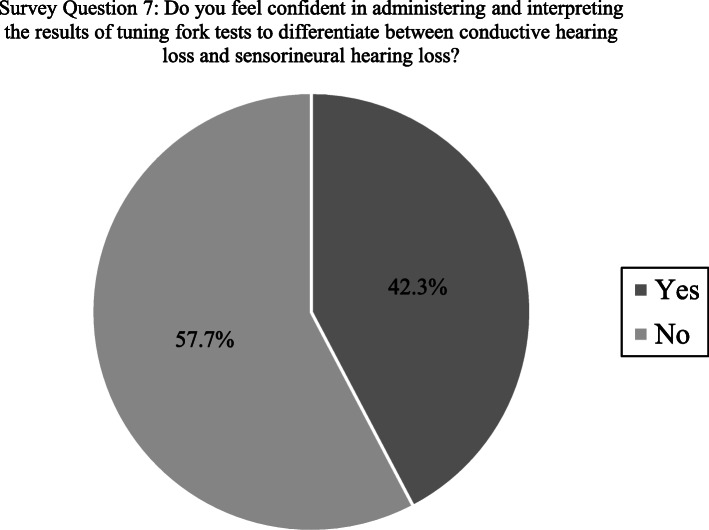
Participant responses that may explain their usage of tuning fork tests in the evaluation of unilateral, sudden-onset hearing loss

If presented with acute or sudden-onset hearing loss in just one ear, 73.1% of participants (*n* = 38) reported that they would attempt to differentiate between conductive and sensorineural hearing loss whereas 26.9% would not (*n* = 14). Regarding the use of tuning forks, 63.5% of participants (*n* = 33) reported using tuning fork tests to differentiate between conductive and sensorineural hearing loss but fewer reported feeling confident administering and interpreting the results of these tests (42.3%; *n* = 22) (Fig. 1). The majority of participants reported that they did not feel comfortable interpreting results of an audiological evaluation (i.e. audiogram) to differentiate between a conductive hearing loss and sensorineural hearing loss (69.2%; *n* = 36) (Table 2).

**Fig. 2:**
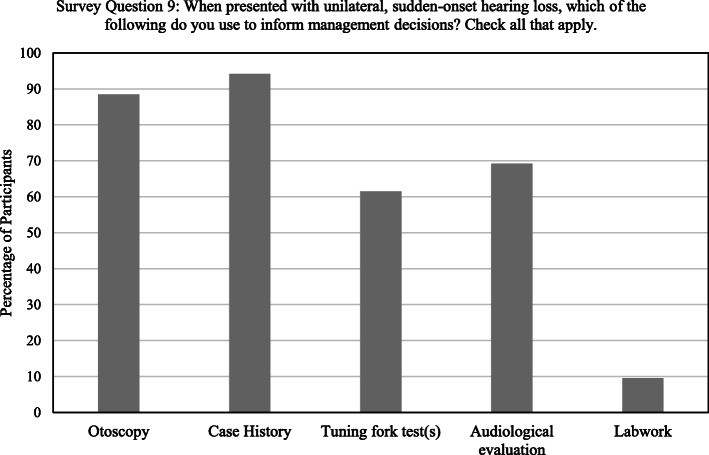
Participant responses indicating the various means utilized for informing management decisions regarding unilateral, sudden-onset hearing loss

When presented with unilateral, sudden-onset hearing loss, the case history and otoscopy were reported by most participants, 94.2% (*n* = 49) and 88.5% (*n* = 46), respectively, to be used for informing management decisions. 61.5% would use tuning fork tests (*n* = 32) and 69.2% would use the results of an audiological evaluation (n = 36) (Fig. 2).

**Fig. 3:**
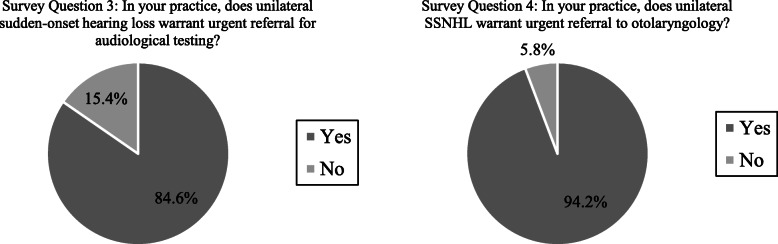
Responses that reflect participants’ understanding of sudden sensorineural hearing loss as a medical emergency

When presented with suspected unilateral SSNHL, 88.5% of participants (n = 46) would refer for audiological evaluation and 80.8% (*n* = 42) would refer for an otolaryngology consultation. Almost all participants (94.2%; n = 49) reported that in their practice, unilateral SSNHL warrants urgent referral to otolaryngology. 84.6% of participants (*n* = 44) reported that in their practice, unilateral sudden-onset hearing loss warrants urgent referral for audiological testing (Fig. 3).

**Fig. 4:**
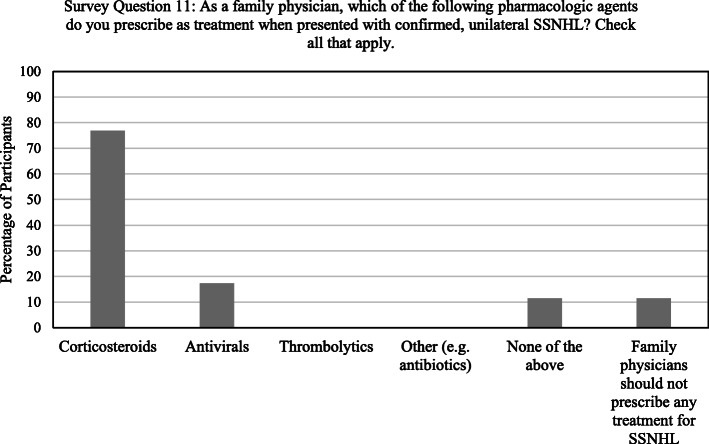
Responses reflecting participants’ knowledge and practices pertaining to the treatment of unilateral, sudden sensorineural hearing loss

75% of participants (*n* = 39) would prescribe corticosteroids for suspected, unilateral SSNHL, even prior to confirmation with audiological testing. 11.5% (*n* = 6) would prescribe corticosteroids and antivirals. 25% (*n* = 13) would not prescribe any pharmacologic agents when presented with suspected SSNHL (Table 2). If presented with confirmed, unilateral SSNHL, 76.9% of participants (*n* = 40) would prescribe corticosteroids and 17.3% (*n* = 9) would prescribe corticosteroids with antivirals. 23.1% of participants (*n* = 12) would not prescribe any pharmacologic agents for confirmed SSNHL, of which six (11.5%) responded that family physicians should not prescribe any treatment. None of the participants reported prescribing thrombolytics, vasodilators or other pharmacologic agents for either suspected or confirmed SSNHL (Fig. 4).

As part of the counselling provided to patients presenting with unilateral SSNHL, 53.8% of participants (*n* = 28) reported including possible causes of the hearing loss. 50% (*n* = 26) include available treatment options and the associated risks/benefits, 25% (*n* = 13) include the impact of the hearing loss on quality of life and 11.5% (*n* = 6) include rehabilitation options, such as the use of amplification. Almost half of participants (42.3%; *n* = 22) responded that they do not provide counselling to patients presenting with SSNHL as they are rarely certain of the diagnosis initially. However, of these 22 participants, four also selected additional response options indicating which topics they would include if they were to provide counselling (Table 2).

## Discussion

The objective of this study was to gain insight into the current knowledge and practice trends pertaining to the diagnosis and management of SSNHL among family physicians in Canada. Our results suggest the majority of family physicians who participated recognize SSNHL is a medical emergency requiring urgent care and would make appropriate referral and treatment decisions if presented with it. However, when presented with undifferentiated, sudden-onset hearing loss, fewer participants utilize all possible means to immediately determine whether the loss is conductive or sensorineural in nature, an important first step in SSNHL management that can potentially impact patient outcomes.

The majority of family physician who participated in our study make appropriate referral decisions when presented with suspected unilateral SSNHL, but a small minority order unnecessary tests. 17.3 and 15.4% of participants order labwork and CT scans, respectively. According to recommendations put forth by the otology/neurotology subspecialty group within the Canadian Society of Otolaryngology – Head and Neck Society, as well as the Clinical Practice Guideline (CPG) for sudden hearing loss established by the American Academy of Otolaryngology – Head and Neck Surgery Foundation (AAO-HNSF), both are unnecessary in the initial evaluation of SSNHL. Results of routine blood work are likely to be normal and even if abnormal, would not change initial management. There is also the risk that false-positives may lead to additional unnecessary testing. CT scans lack the sensitivity to detect retrocochlear pathology and expose patients to radiation. MRI scans of the brain, brainstem and internal auditory canal with gadolinium should be considered instead [43, 51].

The responses to two of the study questions indicate that most of the family physicians who participated understand SSNHL is a time-sensitive emergency requiring prompt medical attention. First, a large majority of participants indicated unilateral, sudden-onset hearing loss would warrant urgent audiological evaluation. This is consistent with AAO-HNSF guidelines, which recommends confirming the diagnosis of SSNHL through audiometry as soon as possible after the onset of symptoms [43]. Early diagnosis allows for initiation of treatment sooner, which increases the likelihood of therapeutic effectiveness. Second, nearly all participants indicated that unilateral SSNHL would warrant an urgent referral to otolaryngology. An otolaryngologist can establish an appropriate management plan, initiate the use of corticosteroids and arrange additional forms of treatment, such as intratympanic steroid therapy and/or hyperbaric oxygen therapy, if necessary.

Urgent referral to otolaryngology is contingent on whether the referring physician can recognize SSNHL at the time of initial presentation, which includes ruling out conductive hearing loss (CHL). According to the AAO-HNSF, a crucial first step for clinicians presented with sudden hearing loss is to differentiate between conductive and sensorineural hearing loss (SNHL), as each requires significantly different management [43]. Nearly three-quarters of participants indicated they attempt to do so when presented with unilateral, acute or sudden-onset hearing loss, primarily through otoscopy and a case history. While these are important and necessary components in the evaluation of sudden hearing loss, they have limitations. Patients may not always provide reliable details regarding their hearing loss and common causes of CHL, such as middle ear effusion and cerumen impaction, can have the same symptoms of tinnitus and aural fullness experienced with SSNHL [52, 53]. Otoscopic examination may not always be reliable for determining the nature of a hearing loss either. The tympanic membrane almost always appears normal in SSNHL, [54] but can in some instances of CHL as well [55, 56]. In either case, information obtained from otoscopy will be limited if view of the ear canal or tympanic membrane is obstructed with cerumen.

Another means of differentiating CHL from SNHL recommended in the initial evaluation of unilateral, sudden hearing loss is the use of tuning fork tests (TFTs), specifically the Weber and Rinne [43, 57, 58]. The Weber test can be used to determine whether a unilateral hearing loss is sensorineural or conductive in nature, and can be highly sensitive at detecting the affected ear in individuals with SSNHL [59]. In the case of a unilateral sensorineural hearing loss, the findings of the Weber test can be verified with the Rinne, by indicating the absence of CHL in the affected ear [60]. Although clearly useful, only 63.5% of participants indicated using TFTs to differentiate between CHL and SNHL, and compared to otoscopy or a case history, far fewer participants indicated using TFTs to inform management decisions when presented with unilateral, sudden-onset hearing loss. Our data also indicates many participants rely on audiological evaluation to inform management decisions in these instances, suggesting they might wait for test results before making an urgent referral to otolaryngology. While the Weber and Rinne tests do not substitute for formal audiological assessment, they can save time and provide family physicians with the immediate information needed to prompt such urgent action. Furthermore, primary care settings may lack audiological testing capabilities so even urgent referral for testing would require patients to travel elsewhere, further delaying diagnosis and treatment.

Confirming the sensorineural nature of a sudden hearing loss can also provide the rationale for a family physician to initiate immediate use of corticosteroids, prior to consultation with otolaryngology, thereby increasing the chance of hearing recovery. 76.9% of participants indicated they prescribe corticosteroids when presented with confirmed, unilateral SSNHL. This figure suggests most family physicians who participated correctly understand corticosteroids can be effective for the initial treatment of SSNHL. This is reassuring considering more than half of participants indicated patients referred to otolaryngology for sudden-onset, unilateral hearing loss waited one to four weeks before being seen, a delay in treatment that could reduce or eliminate the likelihood of recovery. However, the initiation of corticosteroid treatment, just like urgent referral to otolaryngology, depends on whether the referring physician recognizes the sensorineural nature of the presented loss. TFTs, along with otoscopy and a thorough case history, can and should be used by family physicians to make this determination. The relatively limited use of TFTs among our participants may be correlated with their low confidence levels in administering and interpreting such tests. One possible explanation offered by our data is that the family physicians who participated do not encounter sudden hearing loss often enough to use these tests regularly, making it difficult to maintain proficiency. If so, family physicians can use the Hum test instead, a simple and valid alternative to the Weber that does not require a tuning fork [61].

Although the majority of participants indicated they prescribe corticosteroids for the treatment of SSNHL, the remaining utilize an alternate pharmacological approach or none at all. Nine participants indicated they prescribe antivirals in addition to corticosteroids even though previous review and analysis of the literature did not find any statistically significant benefit with their use [62, 63]. Antivirals should not be prescribed as treatment for SSNHL [51], especially given the side effects reported with their use, which include nausea, vomiting, photosensitivity and in rare instances, reversible neurologic reactions [43]. Approximately one-fifth of participants indicated they do not prescribe any pharmacologic agents for even confirmed SSNHL. Although our study is unable to provide a definitive explanation, their position may be due to concern for the possible side effects associated with systemic corticosteroid therapy. Short-term corticosteroid use can cause acne, myopathy, hyperglycemia, hypertension, pancreatitis and psychological disturbances such as mood swings and insomnia, but these typically resolve as treatment begins to taper [58, 64, 65]. Serious side effects and adverse events, such as osteonecrosis, are rare and more likely with long-term use of corticosteroids, not the short 10- to 14-day courses typical for treatment of SSNHL [66–68]. A small minority of participants indicated that family physicians should not prescribe any treatment at all. Perhaps these individuals believe treatment decisions would be best made by otolaryngologists with specialized knowledge and training. Another possibility some participants do not prescribe any treatment for SSNHL is a belief the hearing loss may spontaneously resolve. Although the literature reveals 32 to 65% of cases spontaneously recover, [16, 69] these figures are considered overestimations [43]. Even if not, considering the significant consequence of permanent hearing loss, family physicians should prescribe corticosteroids as initial treatment for SSNHL despite associated risks. The exception would be for patients with contraindications that include diabetes, labile hypertension, glaucoma, tuberculosis, peptic ulcer disease and prior psychiatric reactions to corticosteroids [43, 70, 71].

Our study has several limitations. Despite the several thousand members in the two Facebook groups solicited for our survey, our response yield was low with only 52 participants. Given the small sample size, lack of participants from some provinces and territories, and with the majority of participants having less than five years of practice experience, the responses obtained may not necessarily be representative of the knowledge and management practices of all family physicians in Canada. We are also unable to verify participants’ responses to objective questions (e.g. number of patients presenting with sudden, unilateral hearing loss) which may be inaccurate due to memory and implicit or explicit personal biases. There is also likely the influence of selection bias by utilizing exclusively an electronic survey marketed only to specific discussion groups on a single social media platform. Finally, there is a possibility certain survey questions were susceptible to response bias due to either prior knowledge of the study’s premise (e.g. Q3 In your practice, does unilateral, sudden-onset hearing loss warrant referral for audiological testing?), or wording used (e.g. Q10 As a family physician, which of the following pharmacologic agents do you prescribe as treatment when presented with suspected unilateral SSNHL, prior to confirmation with audiological testing?). When answering these questions, participants may be drawing on their knowledge of how to manage confirmed SSNHL, rather than their actual experience in clinic when a diagnosis is not yet known. If so, responses may overestimate how often family physicians make appropriate management decisions in those situations.

Despite these limitations, the study’s findings reveal opportunities to enhance the knowledge and management practices of PCPs with regards to SSNHL. Continuing Medical Education with a focus on how to administer and interpret TFTs, which diagnostic tests to order or avoid, and appropriate use of therapeutic options could benefit family physicians and help them provide better patient care. Otolaryngologists can and should assume a primary role in providing this education. Formal learning experiences, such as seminars or conference workshops, and dissemination through publications could both be effective means of doing so. To ensure patients receive treatment in a timely manner, PCPs and otolaryngologists should establish conventions for communicating SSNHL during the referral process so that urgent priority can be given.

## Conclusions

In conclusion, the majority of family physicians who participated in our study would make appropriate referral and treatment decisions in the management of SSNHL, recognizing it is a medical emergency requiring urgent care. The majority of participants would prescribe corticosteroids for SSNHL, which is the established standard for initial treatment, but not all do. Further research is needed to understand the rationale for this alternative approach and identify possible gaps in knowledge or improve clinical protocols. Regardless, expeditious referral to otolaryngology or the initiation of corticosteroid therapy is contingent upon the family physician being able to first determine whether the sudden loss of hearing is conductive or sensorineural in nature. TFTs are a quick and reliable means of doing so yet under-utilized by the participants in our study. The majority reported a lack of confidence administering and interpreting the results of TFTs, which may be correlated with their limited use. Our study reveals opportunities for otolaryngologists to provide PCPs with education focusing on the evaluation and treatment of SSNHL that can potentially improve patient outcomes.

## Supplementary Information

**Additional file 1.** Survey. A copy of the study survey.

## Data Availability

All data generated or analyzed during this study are included in this published article [and its supplementary information files].

## Abbreviations

SSNHL: Sudden Sensorineural Hearing Loss
PCP: Primary Care Physician
CPG: Clinical Practice Guideline
AAO-HNSF: American Academy of Otolaryngology – Head and Neck Surgery Foundation
CHL: Conductive Hearing Loss
SNHL: Sensorineural Hearing Loss
TFTs: Tuning Fork Tests
