# Supplementary material for: Management of sudden sensorineural hearing loss among primary care physicians in Canada: a survey study
Source: J Otolaryngol Head Neck Surg. 2021 Apr 1;50:22. doi: 10.1186/s40463-021-00498-x (PMC8015047; doi:10.1186/s40463-021-00498-x)
Supplement: Supplementary file 1 — Additional file 1. Survey. A copy of the study survey. [file 40463_2021_498_MOESM1_ESM.pdf]

SURVEY

There are a total of 18 multiple-choice questions divided into two sections that pertain to: 1) trends in Sudden Sensorineural Hearing Loss (SSNHL) management; 2) demographic information. When answering the questions, unless specifically indicated as SSNHL, “hearing loss” refers to a perceived hearing impairment that has not yet been differentiated as either conductive or sensorineural in nature.

Questions Assessing Trends in SSNHL Management

1. According to *your* definition, sudden sensorineural hearing loss (SSNHL) is defined as hearing loss that can develop over a period of:
   a. Less than 24 hours
   b. 48 hours
   c. 72 hours
   d. 7 days

2. Which of the following referrals do you make upon presentation of suspected unilateral SSNHL? Check all that apply.
   a. Labwork
   b. CT
   c. MRI
   d. Audiological evaluation
   e. Otolaryngology consultation
   f. Neurology consultation
   g. Emergency Department

3. In your practice, does unilateral, sudden-onset hearing loss warrant *urgent* referral for audiological testing?
   a. Yes
   b. No

4. In your practice, does unilateral SSNHL warrant *urgent* referral to otolaryngology?
   a. Yes
   b. No

5. When presented with unilateral, acute or sudden-onset hearing loss, do you attempt to differentiate between conductive and sensorineural hearing loss?
   a. Yes
   b. No

6. Do you use tuning fork tests to differentiate between conductive and sensorineural hearing loss?
   a. Yes
   b. No

7. Do you feel confident in administering and interpreting the results of tuning fork tests to differentiate between conductive hearing loss and sensorineural hearing loss?
    a. Yes
    b. No

8. Do you feel comfortable interpreting a formal audiogram to differentiate between a conductive hearing loss and sensorineural hearing loss?
    a. Yes
    b. No

9. When presented with unilateral, sudden-onset hearing loss, which of the following do you use to inform management decisions? Check all that apply.
    a. Otoscopy
    b. Case history
    c. Tuning fork test(s)
    d. Audiological evaluation
    e. Labwork

10. As a family physician, which of the following pharmacologic agents do you prescribe as treatment when presented with *suspected* unilateral SSNHL, prior to confirmation with audiological testing? Check all that apply.
    a. Corticosteroids
    b. Antivirals
    c. Thrombolytics
    d. Vasodilators
    e. Other (e.g. antibiotics)
    f. None of the above
    g. I do not prescribe any pharmacologic agents when presented with suspected SSNHL

11. As a family physician, which of the following pharmacologic agents do you prescribe as treatment when presented with *confirmed*, unilateral SSNHL? Check all that apply.
    a. Corticosteroids
    b. Antivirals
    c. Thrombolytics
    d. Vasodilators
    e. Other (e.g. antibiotics)
    f. None of the above
    g. Family physicians should not prescribe any treatment for SSNHL

12. Which of the following topics *do you* include as part of counselling to patients presenting with unilateral SSNHL? Check all that apply.
    a. Possible causes
    b. Available treatment options and associated risks/benefits
    c. Impact on Quality of Life
    d. Rehabilitation options (e.g. hearing aids)

e. None of the above
f. I do not counsel patients presenting with SSNHL as I am rarely certain of the diagnosis upon initial presentation

<u>Demographic Questions</u>

13. Years in practice
    a. Less than 5 years
    b. 5 – 10 years
    c. 11 – 15 years
    d. 16 – 20 years
    e. More than 20 years

14. The geographical region in which you primarily serve patients would be considered:
    a. Urban
    b. Suburban
    c. Rural

15. The setting in which you primarily practice would be considered:
    a. Solo practice
    b. Walk-in clinic
    c. Urgent Care or Emergency Department
    d. Non-Academic Group or Team
    e. Academic Group or Team

16. In the past 6 months, number of patients presenting to you with complaints of unilateral, acute or sudden-onset hearing loss, *not* as a result of cerumen impaction:
    a. Fewer than 5
    b. 5 – 10
    c. 11 – 15
    d. 16 – 20
    e. More than 20

17. In the past 12 months, typical wait-time for your patients to be seen by an otolaryngologist, referred for unilateral, sudden-onset hearing loss:
    a. 1 week or less
    b. 1 – 4 weeks
    c. 1 – 3 months
    d. 3 – 6 months
    e. Greater than 6 months
    f. Greater than 12 months

18. The province/territory in which you primarily practice:
    a. British Columbia
    b. Alberta
    c. Saskatchewan

d. Manitoba
e. Ontario
f. Quebec
g. New Brunswick
h. Nova Scotia
i. Prince Edward Island
j. Newfoundland and Labrador
k. Yukon
l. Northwest Territories
m. Nunavut
